# Using interferon-beta to combat cancer stem cell properties in triple negative breast cancer

**DOI:** 10.18632/oncoscience.438

**Published:** 2018-06-28

**Authors:** Mary R. Doherty, Mark W. Jackson

**Affiliations:** Department of Pathology, Case Western Reserve University, School of Medicine, Cleveland, OH 44106, USA; bCase Comprehensive Cancer Center, Case Western Reserve University, School of Medicine, Cleveland, OH 44106, USA

**Keywords:** interferon-beta, triple-negative breast cancer, cancer stem cells, cellular plasticity

Triple Negative Breast Cancer (TNBC) is the most lethal form of breast cancer. Currently, targeted therapies for TNBC are lacking, leaving cytotoxic chemotherapy and radiotherapy as the standards of care. Emerging evidence now suggests that highly aggressive, metastatic and therapy-resistant TNBC tumors harbor Cancer Stem Cell (CSC) plasticity, an ability to shift between CSC and non-CSC states, often coincident with epithelial-mesenchymal (E-M) plasticity (in which, non-CSC retain epithelial markers and CSC acquire mesenchymal markers) [1]. Acquisition of CSC properties can occur spontaneously during cellular transformation, as evidenced by the gain of CSC and mesenchymal markers (CD44, NANOG, VIMENTIN, SNAIL1, SLUG) and an increase in CSC-associated behaviors (tumor sphere formation, increased migration and invasion, tumor-initiating capacity) [2]. Importantly, we have found that non-CSC can be induced into a CSC-like state following exposure to tumor-associated cytokines often elevated in the breast tumor microenvironment (TME) of patients with increased risk of tumor recurrence and poor prognosis. Cytokine-induced CSC retain plasticity, as either pharmacologic or genetic inhibition of cytokine receptor/effector signaling is sufficient to revert cells back into a more differentiated, non-CSC state [3-4].

Other recent evidence has shown that cytotoxic chemotherapy can induce the expression of CSC markers and behaviors associated with CSC in various cancer types (reviewed in reference [1]). For example, acute exposure to Adriamycin or Taxanes drives the adaptive emergence of therapy-resistant, CD44-High CSC in both breast tumor explants as well as breast cancer cell lines [5]. These findings suggest that cellular plasticity may be a critical, adaptive survival mechanism utilized by tumor cells to evade therapy-induced killing, ultimately driving tumor recurrence. Additional studies also support the idea that tumor cells can be “reprogrammed” by chemotherapy, rather than simply selecting for a subset of pre-existing CSC [6]. Therefore, identifying the pathways that regulate CSC plasticity could lead to the development of a critically needed targeted therapy for TNBC.

**Figure 1:**
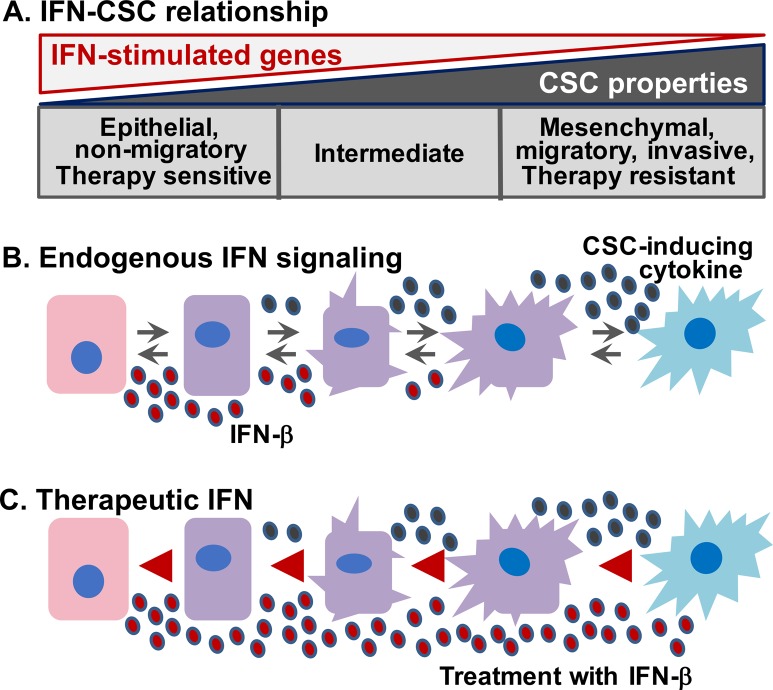
IFN represses CSC properties in TNBC by driving tumor cell differentiation and a non-CSC state **(A)** Human Mammary Epithelial Cells (HMEC) that acquire Mesenchymal/CSC properties lose IFN-β stimulated gene expression. **(B)** Elevated endogenous IFN-β promotes tumor cell differentiation to a less aggressive Epithelial/non-CSC state. **(C)** Treatment with IFN-β has therapeutic potential as a CSC-targeted therapy for TNBC by differentiating CSC into less aggressive Epithelial/non-CSCs.

In our recent work, we utilized a Human Mammary Epithelial Cell (HMEC) transformation model which mirrors the immune-activated sub-type of TNBC. Immune activated TNBC exhibit enhanced Interferon/Signal Transducer of Activated Transcription (IFN/STAT1) signaling and gene expression and elevated numbers of tumor-infiltrating lymphocytes (TILs). Patients with immune-activated TNBC have improved therapeutic responses and better overall survival when compared to other TNBC sub-types [7]. Importantly, as transformed HMEC acquire a mesenchymal/CSC phenotype (increased migration and tumor sphere-forming capacity), they lose IFN/STAT1-stimulated gene (ISG) expression (Figure 1A). Patients with immune-repressed TNBC are characterized by reduced IFN/STAT1 signaling and gene expression and reduced TILs, correlating with increased risk of tumor recurrence and decreased overall survival. Importantly, we found that treatment with non-cytotoxic, non-cytostatic doses of IFN-β (but not IFN-γ) could induce ISG expression, resulting in the differentiation of CSC into the less aggressive epithelial state with increased CD24 expression and reduced mesenchymal markers (VIMENTIN and SLUG). The cells also had reduced migration and tumor sphere formation capabilities. We validated our findings using clinical specimens, showing that the presence of an IFN-β/STAT1 gene expression signature negatively correlated with the expression of a CSC gene signature, and positively correlated with TILs and improved patient outcome [2].

Taken together, our study provides two key insights into how IFN/STAT1 signaling impacts patients with TNBC. First, our findings begin to explain why the presence of endogenous IFN/STAT1 signaling correlates with improved patient outcomes. IFN is well-known for its immune-modulating functions, so a TME with increased IFN would undoubtedly have elevated immune cell infiltration (likewise, a TME with elevated immune cell infiltration would have increased IFN levels). Our studies show, that beyond the impact on the immune cells, IFN-β impacts tumor cell differentiation status directly, imparting a more differentiated, less aggressive phenotype to the cancer cells (Figure 1B). Second, our study highlights the potential use of IFN-β as a CSC-targeted therapy (Figure 1C). The ability of IFN-β to induce differentiation in Mesenchymal/CSC to a less aggressive, non-stem state suggests that, if IFN-β can be targeted to immune-repressed TNBC, it could mitigate the aggressive properties associated with the lack of endogenous IFN/STAT1 signaling. We envision that IFN-β would not only induce the differentiation of CSC, but also prevent the de-differentiation of non-CSC into CSC that occurs during treatment with chemotherapies. Our studies are now combining IFN-β treatment with chemotherapy to define whether IFN can oppose the adaptive emergence of therapy-resistant CSC.
